# Development and evaluation of a machine learning model to predict unplanned readmission risk in patients with ulcerative colitis

**DOI:** 10.3389/fmed.2026.1712846

**Published:** 2026-01-27

**Authors:** Tianqi Wang, Yujie Zhao, Xiaobin Zhao, Jiaqi Zhu, Junyi Zhan, Dongli Wang

**Affiliations:** 1 First Clinical Medical College, Shandong University of Traditional Chinese Medicine, Jinan, Shandong, China; 2 Department of Gastroenterology, Affiliated Hospital of Shandong University of Traditional Chinese Medicine, Jinan, Shandong, China; 3 Department of Gastroenterology, Hangzhou Hospital of Traditional Chinese Medicine, Hangzhou, Zhejiang, China

**Keywords:** machine learning, online platform, predictive model, ulcerative colitis, unplanned readmission

## Abstract

**Objective:**

Ulcerative colitis (UC), a chronic inflammatory bowel disease marked by recurrent flares and remissions, often necessitates repeated hospitalization owing to disease variability. However, commonly used risk-scoring systems have limited predictive accuracy for hospital readmission. This study aimed to develop and validate a machine learning (ML)-based model to predict the risk of unplanned readmission within 1 year in patients with UC.

**Methods:**

Unplanned readmission within 1 year was defined as an endpoint event, and a predictive model was developed using a retrospective cohort (*n* = 324) and externally validated using an independent prospective cohort (*n* = 137). Demographic characteristics, medical history, medication use, clinical symptoms, laboratory findings, and endoscopic data were integrated as input variables. The optimal feature subset was selected using Recursive Feature Elimination (RFE), and eight ML models were constructed. All models were optimized via five-fold cross-validation, and the best-performing model was selected as the final predictive tool and was subjected to external validation. Shapley additive explanation plots were used to interpret the predictive model.

**Results:**

The RFE algorithm identified five critical predictors: C-reactive protein, erythrocyte sedimentation rate, red blood cell count, increased frequency of bowel movements, and platelet count. All ML models achieved an AUC above 0.75 in the training cohort, demonstrating their robust predictive capability. The random forest (RF) model consistently outperformed the others across the training, internal validation, and external validation cohorts, with AUCs of 0.936, 0.815, and 0.813, respectively, reflecting excellent stability and generalization. Building upon the RF model, an online risk prediction platform was developed to estimate the probability of unplanned readmission in patients with UC.

**Conclusion:**

The RF-based model showed strong predictive accuracy for assessing the 1-year risk of unplanned readmission in UC patients. The corresponding web-based risk calculator offers clinicians a valuable tool for personalized risk evaluation and enhanced patient management.

## 1 Introduction

Ulcerative colitis (UC) is a chronic and relapsing inflammatory bowel disease that predominantly involves the colonic mucosa, with its etiology strongly linked to immune dysregulation, environmental exposure, and genetic predisposition (1, 2). As of 2023, UC affects approximately 5 million people globally, and its incidence continues to rise, with Europe showing the highest rates, Norway reporting 505 cases per 100,000 population (1, 3). The advent of biologics and immunomodulators in recent years has led to substantial improvements in disease control for certain patients with UC, especially those with moderate to severe activity or poor response to conventional treatments (4, 5). Nonetheless, UC remains a disease characterized by a protracted course and high relapse rate, posing a considerable burden on patient’s quality of life and healthcare systems (6).

The readmission rate (7) serves as a key metric for assessing disease control and management quality in UC. Evidence indicates that patients with UC face a heightened risk of readmission during disease flares and treatment transitions, highlighting the critical value of timely intervention within 1 year (8, 9). Consequently, accurate prediction of readmission risk enables clinicians to identify high-risk individuals and implement tailored follow-up and intervention plans, ultimately enhancing disease control and reducing the burden of rehospitalization.

While current prognostic models for UC offer preliminary insight into unplanned readmission risk, they are limited by incomplete incorporation of clinical variables, such as laboratory results, medication profiles, and comorbidities, as well as by the lack of external validation and cross-model comparative analysis (10, 11). Thus, a practical and widely applicable predictive tool is urgently needed to assist in managing the risk of readmission among patients with UC.

In summary, this study sought to leverage multiple machine learning (ML) algorithms and comprehensive clinical data to build and externally validate predictive models, ultimately developing an online tool for assessing 1-year unplanned readmission risk in patients with UC to enhance their clinical management.

## 2 Methods

### 2.1 Study design and subjects

The training dataset comprised a retrospective cohort of 324 patients with UC who were hospitalized at the Affiliated Hospital of Shandong University of Traditional Chinese Medicine between September 2015 and September 2020. The external validation cohort included 137 patients with UC with prospectively collected data who were hospitalized at the same center between October 2020 and October 2023. All patients underwent 1-year follow-up using telephone interviews, outpatient evaluations, and inpatient medical records.

The inclusion criteria were as follows: (1) diagnosis in accordance with the American College of Gastroenterology (ACG) guidelines (12); (2) age ≥ 18 years and ≤ 80 years, regardless of sex.

The exclusion criteria were as follows: (1) presence of severe dysfunction of the heart, liver, or kidneys; serious infections; hematologic diseases; or malignancies; (2) concurrent autoimmune diseases such as rheumatoid arthritis or systemic lupus erythematosus; (3) a history of colectomy in patients with UC; (4) substantial missing data in medical records (> 15%); and (5) poor treatment compliance or loss to follow-up.

This study complied with the Declaration of Helsinki and was approved by the Ethics Committee of the Affiliated Hospital of Shandong University of Traditional Chinese Medicine (Approval No. 2024-152-ky). All participants or their legal guardians provided informed consent by signing a consent form.

### 2.2 Outcomes

Endpoint events were defined as unplanned readmissions within 1 year. Unplanned readmission is defined as an unexpected hospitalization resulting from sudden changes in a patient’s condition or inadequate management, occurring outside of scheduled routine follow-up monitoring, enteral nutrition therapy, medication infusion, or other planned interventions (13). Each case of suspected unplanned readmission was independently assessed by two senior physicians, each with over 10 years of clinical experience. Any discrepancies in the evaluation were resolved through discussion until a consensus was reached.

### 2.3 Candidate predictors and data collection

Candidate predictors included only variables collected at the time of initial hospital admission. The following data were collected: (1) demographic factors, including age, sex, smoking history, and alcohol use; (2) past medical history, such as hypertension, diabetes, cardiovascular disease, and anemia; (3) medication regimen, including 5-aminosalicylic acid drugs, corticosteroids, immunosuppressants, and probiotics; (4) clinical manifestations, such as increased bowel frequency; (5) laboratory tests, including blood counts, liver and kidney function, electrolytes, and coagulation; (6) endoscopic data, including colonoscopy results and mucosal histology; and (7) clinical assessment tools, primarily the Mayo score (12), the Mayo Endoscopic Subscore (MES) (14), the Degree of Ulcerative Colitis Burden of Luminal Inflammation (DUBLIN) score (15), and the Ulcerative Colitis Endoscopic Index of Severity (UCEIS) (16). Two data managers independently collected and cross-checked all clinical data to ensure accuracy and consistency.

### 2.4 Statistical analysis and model construction

All statistical analyses were performed using R software (version 4.3.2, https://www.R-project.org). Multiple imputation was used to address missing data, and variables with over 15% missingness were excluded from the analysis. Descriptive statistics are presented as *n* (%), mean ± SD, or median (Q1, Q3), as appropriate. Group comparisons were conducted using Student’s *t*-test for normally distributed continuous variables and the Mann–Whitney U test for those not normally distributed. Categorical data were analyzed using Pearson’s χ^2^ test or Fisher’s exact test, depending on the expected frequencies. Statistical significance was set at *p* < 0.05 (two-tailed).

The optimal feature subset was selected using Recursive Feature Elimination (RFE) and applied to the training and validation of each ML model. Eight different ML models were trained and tested, including Random Forest (RF), Decision Tree (DT), K-Nearest Neighbors (KNN), Light Gradient Boosting Machine (Light GBM), Logistic Regression (LR), Multilayer Perceptron (MLP), Support Vector Machine (SVM), and Extreme Gradient Boosting (XG Boost). The training dataset was used for model development, and five-fold cross-validation was performed for hyperparameter tuning and internal validation. An independent prospective cohort was used for external validation, and the best-performing model was selected as the final model. The model performance was evaluated using metrics including the area under the receiver operating characteristic curve (AUC), accuracy, F1 score, recall, precision, sensitivity, and specificity. In addition, the optimal model was compared with the Mayo score, the MES, the DUBLIN score, and the UCEIS in the training and external validation cohorts. The classification performance was assessed using the precision-recall (PR) curve, and the clinical net benefit was evaluated using Decision Curve Analysis (DCA).

Shapley additive explanation (SHAP) was used to analyze the contribution and direction of each predictor. A web-based risk prediction platform was developed based on the optimal model and feature importance of the predictive variables.

## 3 Results

### 3.1 Comparison of clinical characteristics

This study included 461 eligible patients, of whom 324 were assigned to the training cohort and 137 to the validation cohort. Table 1 summarizes the baseline characteristics and missing data patterns of the included patients. After removing variables with missing rates over 15%, 45 variables were included in the statistical analysis (*p* > 0.05). Details of the excluded fecal calprotectin data are presented in Supplementary Table 1. The training and validation cohorts were comparable in terms of major clinical features, with no statistically significant differences. Compared with the training cohort, the external validation cohort showed significantly lower mean corpuscular hemoglobin concentration, mean corpuscular volume, serum potassium, and apolipoprotein A1 levels, but higher mean platelet volume and serum chloride levels (*p* < 0.05).

**Table 1:** Baseline characteristics and outcomes of the training and validation cohort.

Characteristic	Training cohort (*n* = 324)	Missing data in training cohort (%)	Validation cohort (*n* = 137)	Missing data in testing cohort (%)	*P*-value
Demographics					
Age (year)	47 (36–55.75)	0	46 (35–56)	0	0.87
Sex, *n* (%)		0		0	0.736
Female	197 (60.8)		81 (59.1)		
Male	127 (39.2)		56 (40.9)		
Smoking history, *n* (%)	37 (11.4)	0	13 (9.5)	0	0.542
Drinking history, *n* (%)	31 (9.6)	0	11 (8)	0	0.600
Basic diseases					
Hypertension, *n* (%)	27 (8.3)	0	13 (9.5)	0	0.687
Heart disease, *n* (%)	10 (3.1)	0	8 (5.8)	0	0.163
Diabetes, *n* (%)	10 (3.1)	0	7 (5.1)	0	0.292
Anemia, *n* (%)	67 (20.7)	0	22 (16.1)	0	0.251
Endoscopic manifestations					
Clinical typing, *n* (%)		2.7		2.1	0.854
Initial hairstyle	38 (12.0)		17 (12.4)		
Recurrent type	277 (85.5)		117 (85.4)		
Extent of disease, *n* (%)		11.4		2.1	0.138
E1	83 (25.6)		31 (22.6)		
E2	100 (30.9)		50 (36.5)		
E3	104 (32.1)		53 (38.7)		
Colonoscopy examination, *n* (%)		0		0	0.938
Relief period	16 (4.9)		7 (5.1)		
Activation	308 (95.1)		130 (94.9)		
Clinical symptoms					
Increased frequency of bowel movements (based on Mayo score), *n* (%)		0		0	0.885
Normal	93 (20.2)		66 (20.4)		
Mild	123 (26.7)		84 (25.9)		
Moderate	124 (26.9)		86 (26.5)		
Severe	121 (26.3)		88 (27.2)		
Use of medication					
Use of 5-ASA, *n* (%)	268 (82.7)	0	117 (85.4)	0	0.478
Use of GCS, *n* (%)	68 (21)	0	26 (19)	0	0.625
Use of immunosuppressive agents, *n* (%)	13 (4)	0	3 (2.2)	0	0.629
Use of probiotics, *n* (%)	141 (43.5)	0	56 (40.9)	0	0.600
Laboratory tests					
Platelet crit (%)	0.26 (0.22–0.32)	7.41	0.27 (0.22–0.32)	0	0.573
Red cell distribution width standard deviation (fL)	40.9 (39.2–43.68)	7.10	41.75 (39.55–44.3)	0.7	0.135
Eosinophil absolute count (×10^9^/L)	0.13 (0.06–0.22)	10.49	0.12 (0.06–0.2)	0.7	0.483
Platelet distribution width (fL)	11.2 (10–12.6)	7.41	11 (9.9–12.2)	0	0.146
Platelet-larger cell ratio (%)	25.05 (19.7–30.85)	7.41	24.1 (20.15–28.25)	0	0.46
Mean platelet volume (fL)	10 (9.4–10.7)	7.41	9.9 (9.5–10.5)	0	0.522
Platelet count (×10^9^/L)	265 (213–332)	6.48	269 (223–330.5)	0	0.447
Red cell distribution width (%)	12.7 (12.2–14)	7.10	12.6 (12.1–13.5)	0.7	0.199
Mean corpuscular hemoglobin concentration (g/L)	332 (320–343)	6.48	327 (315–338)	0	0.005
Mean corpuscular hemoglobin (pg)	29.6 (27.4–30.9)	6.48	29.6 (27.75–31)	0	0.662
Mean Corpuscular Volume (fL)	87.9 (84.05–91.4)	6.48	89.3 (84.65–92.65)	0	0.022
Packed cell volume (%)	38.5 (34.23–41.68)	6.48	38.8 (34.2–43)	0	0.384
Hemoglobin (g/L)	127.5 (113–140)	6.48	127 (110–142)	0	0.955
Red blood cell (×10^12^/L)	4.31 (3.83–4.68)	6.79	4.34 (3.89–4.78)	0	0.22
Neutrophil granulocyte (×10^9^/L)	3.81 (2.78–5.3)	6.48	3.85 (2.88–5.35)	0	0.483
Mono (×10^9^/L)	0.46 (0.33–0.66)	6.48	0.49 (0.39–0.63)	0	0.368
Lymph (×10^9^/L)	1.585 (1.26–2.08)	6.48	1.65 (1.27–2.05)	0	0.648
White blood cell (×10^9^/L)	5.95 (4.78–7.85)	7.10	6.39 (5.1–8.11)	0	0.197
Albumin (g/L)	39.2 (35.08–42.6)	6.17	38.5 (34.8–42.3)	1.5	0.471
C-reactive protein (mg/L)	6.7 (2.63–19)	9.26	5.9 (1.93–17.5)	0.7	0.296
Na (mmol/L)	139 (138–141)	6.17	140 (138–141)	0.7	0.217
K (mmol/L)	3.96 (3.71–4.16)	6.17	3.85 (3.57–4.15)	0.7	0.02
CL (mmol/L)	105 (103–106)	6.48	105 (103–107)	0.7	0.032
Globularproteins (g/L)	27.4 (24.83–30.78)	6.48	27.7 (25.2–30.1)	1.5	0.948
CA (mmol/L)	2.26 (2.15–2.34)	6.17	2.23 (2.12–2.33)	0.7	0.18
Erythrocyte sedimentation rate (mm/h)	13.5 (7–25.75)	11.11	11 (7–26)	0	0.398
Alanine aminotransferase (U/L)	13 (10–21)	7.72	14 (10–23.75)	0.7	0.272
Aspartate aminotransferase (U/L)	17 (13–22)	7.41	17 (14–21.75)	0.7	0.517
Total bilirubin (μmoI/L)	10.8 (8.3–14.2)	6.48	11.1 (8.25–15.15)	0	0.417
Direct bilirubin (μmoI/L)	2.1 (1.5–2.9)	6.48	2.3 (1.65–3.1)	0	0.100
Immunoglobulin bilirubin binding capacity (μmoI/L)	8.6 (6.83–11.4)	6.48	9 (7.05–13.05)	0	0.158
Apolipoprotein A1 (g/L)	1.28 (1.08–1.58)	7.10	1.22 (1.06–1.41)	0	0.010
Apolipoprotein B (g/L)	0.8 (0.67–1.0)	7.10	0.76 (0.64–0.95)	0	0.152
Prealbumin (mg/L)	204 (160.25–244.75)	6.48	196 (160.5–228.5)	0	0.360
Blood urea nitrogen (mmol/L)	4.14 (3.26–4.97)	6.48	4.08 (3.29–4.89)	0	0.768
Creatinine (μmoI/L)	58 (49–68)	6.48	56 (48–67)	0.7	0.175
Clinical score					
Mayo rating system, *n* (%)		4.3		5.1	0.4
Remission	17 (5.2)		5 (3.6)		
Mild	81 (25)		44 (32.1)		
Moderate	206 (63.6)		79 (57.7)		
Moderate	6 (1.9)		2 (1.5)		
Mayo endoscopic score, *n* (%)		4.6		2.9	0.734
Remission	6 (1.9)		3 (2.2)		
Mild	78 (24.1)		27 (19.7)		
Moderate	143 (44.1)		66 (48.2)		
Moderate	82 (25.3)		37 (27.0)		
DUBLIN score	4 (2–6)	13.3	4 (2–6)	12.4	0.598
UCEIS	3 (1–4)	0	3 (2–4)	0	0.297

5-ASA, 5-Aminosalicylic Acid; GCS, glucocorticosteroid; DUBLIN score, Degree of Ulcerative Colitis Burden of Luminal Inflammation; UCEIS, Ulcerative Colitis Endoscopic Index of Severity. Statistical significance, *p* < 0.05.

### 3.2 Selection of key predictors

RFE was used to identify key features from 45 candidate variables in the training cohort. The highest accuracy (80.61%) was achieved when the model selected an optimal subset of five clinical predictors (Supplementary Figure 1). The five selected features were C-reactive protein (CRP) level, erythrocyte sedimentation rate (ESR), red blood cell count (RBC), increased bowel movement frequency (IFBM, based on the Mayo score), and platelet count (PLT).

### 3.3 Multiple ML model performance

**Figure 1:**
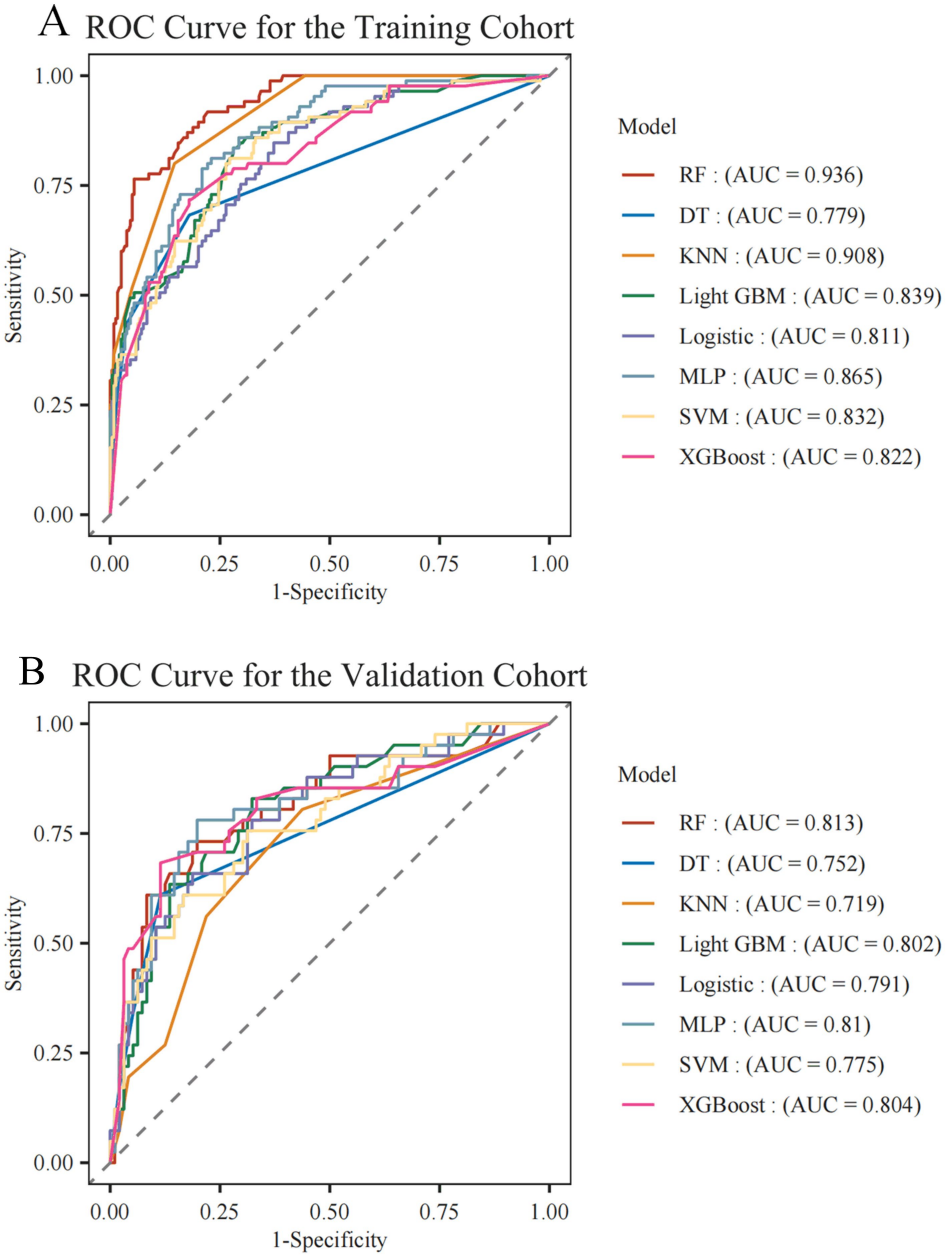
AUC comparison of eight machine learning models in predicting unplanned readmission of UC in **(A)** training cohort and **(B)** validation cohort. DT, decision tree; KNN, *k*-nearest neighbors; Light GBM, light gradient boosting machine; MLP, multilayer perceptron; RF, random forest; SVM, support vector machine, XG Boost, extreme gradient boosting.

In the training dataset, all models, except the DT model (AUC = 0.779), achieved an AUC exceeding 0.80. The RF model exhibited the highest performance, with an AUC of 0.936 (Figure 1A). In internal validation, the RF model continued to demonstrate superior predictive power (AUC = 0.815) via five-fold cross-validation, whereas the remaining models also showed good performance, with AUCs above 0.70. In the external validation cohort, all models yielded AUCs above 0.75, reflecting their strong robustness and generalization capability. Notably, the RF model continued to outperform the others, achieving an AUC of 0.813 (Figure 1B). The RF model consistently outperformed the others across all datasets (training, internal, and external), demonstrating excellent predictive accuracy and clinical utility (Table 2). The calibration plot indicates that the RF model shows reasonably good calibration (Supplementary Figure 2). Accordingly, the RF model was chosen as the final predictive tool for assessing the risk of unplanned readmission in patients with UC.

**Table 2:** Predictive performance of eight machine learning models.

Model	AUC (%)	Accuracy (%)	Sensitivity (%)	Specificity (%)	F1 score (%)	Recall (%)	Precision (%)
Training cohort							
DT	77.9	78.4	82.01	68.24	84.85	82.01	87.89
LR	81.15	68.52	62.76	84.71	74.63	62.76	92.02
RF	93.56	89.81	94.56	76.47	93.2	94.56	91.87
XG Boost	82.21	79.32	82.01	71.76	85.4	82.01	89.09
SVM	83.18	75	72.8	81.18	81.12	72.8	91.58
MLP	86.54	78.09	76.99	81.18	83.83	76.99	92
Light GBM	83.95	75	71.97	83.53	80.94	71.97	92.47
KNN	90.81	83.95	85.36	80	88.7	85.36	92.31
Internal validation							
DT	73.69	77.49	88.52	47.79	85.18	88.52	82.6
LR	77.35	79.33	93.35	39.84	86.9	93.35	81.35
RF	81.47	77.44	91.71	38.56	85.62	91.71	80.55
XG Boost	80.5	80.53	89.91	54.1	87.17	89.91	84.79
SVM	80.46	79.95	98.37	28.05	87.85	98.37	79.42
MLP	79.75	74.39	87.82	36.65	83.37	87.82	79.45
Light GBM	79.51	73.78	100	0	84.82	100	73.78
KNN	80.17	81.16	95.16	43.81	88.05	95.16	82.32
Validation cohort							
DT	75.22	80.29	88.54	60.98	86.29	88.54	84.16
LR	79.07	70.8	68.75	75.61	76.74	68.75	86.84
RF	81.29	81.02	89.58	60.98	86.87	89.58	84.31
XG Boost	80.39	81.75	88.54	65.85	87.18	88.54	85.86
SVM	77.52	70.8	72.92	65.85	77.78	72.92	83.33
MLP	81.05	78.83	82.29	70.73	84.49	82.29	86.81
Light GBM	80.22	71.53	71.88	70.73	77.97	71.88	85.19
KNN	71.88	71.53	78.13	56.1	79.37	78.13	80.65

DT, decision tree; LR, logistic regression; RF, random forest; XG Boost, extreme gradient boosting; SVM, support vector machine; MLP, multilayer perceptron; Light GBM, light gradient boosting machine; KNN, k-nearest neighbors; AUC, area under the receiver operating characteristic curve.

**Figure 2:**
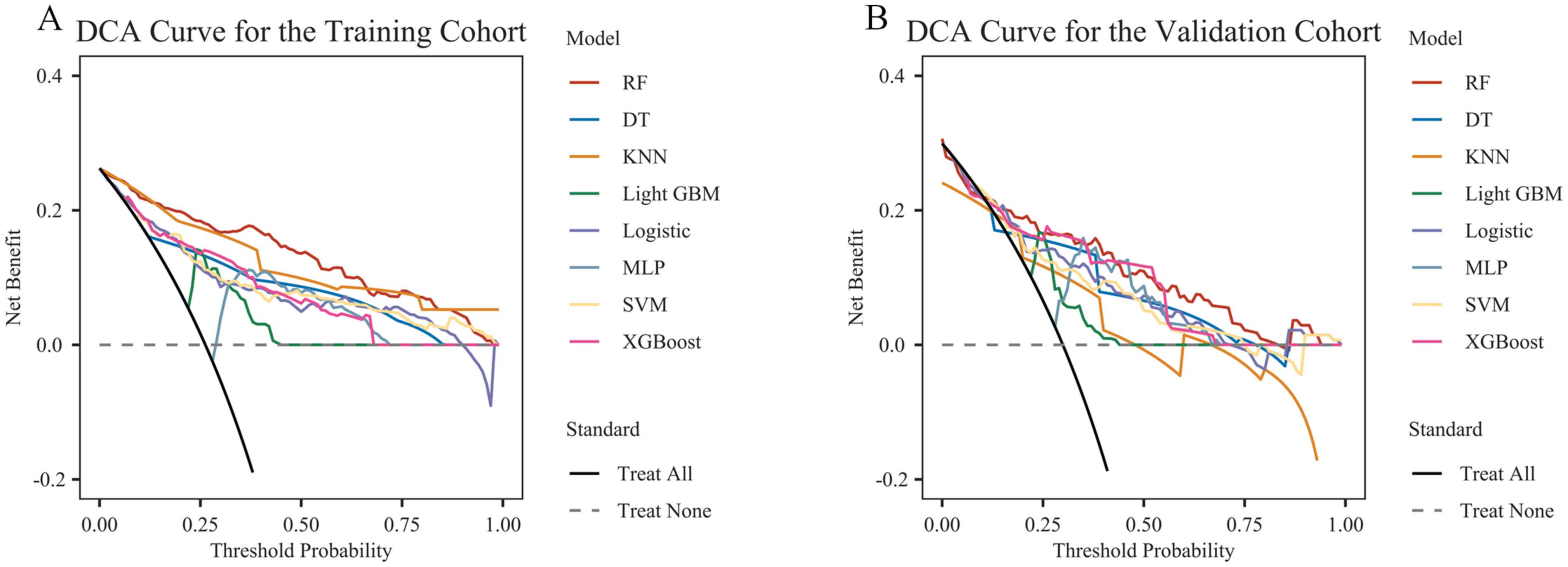
Decision curve analysis (DCA) of different machine learning models in the training cohort **(A)** and external validation cohort **(B)**.

DCA revealed that the model provided positive clinical benefits across a wide range of threshold probabilities in both the training and external validation cohorts (Figure 2). Notably, the RF model showed persistently higher net benefits in both cohorts, especially at lower to intermediate probability thresholds, suggesting greater clinical utility in guiding decision-making.

**Figure 3:**
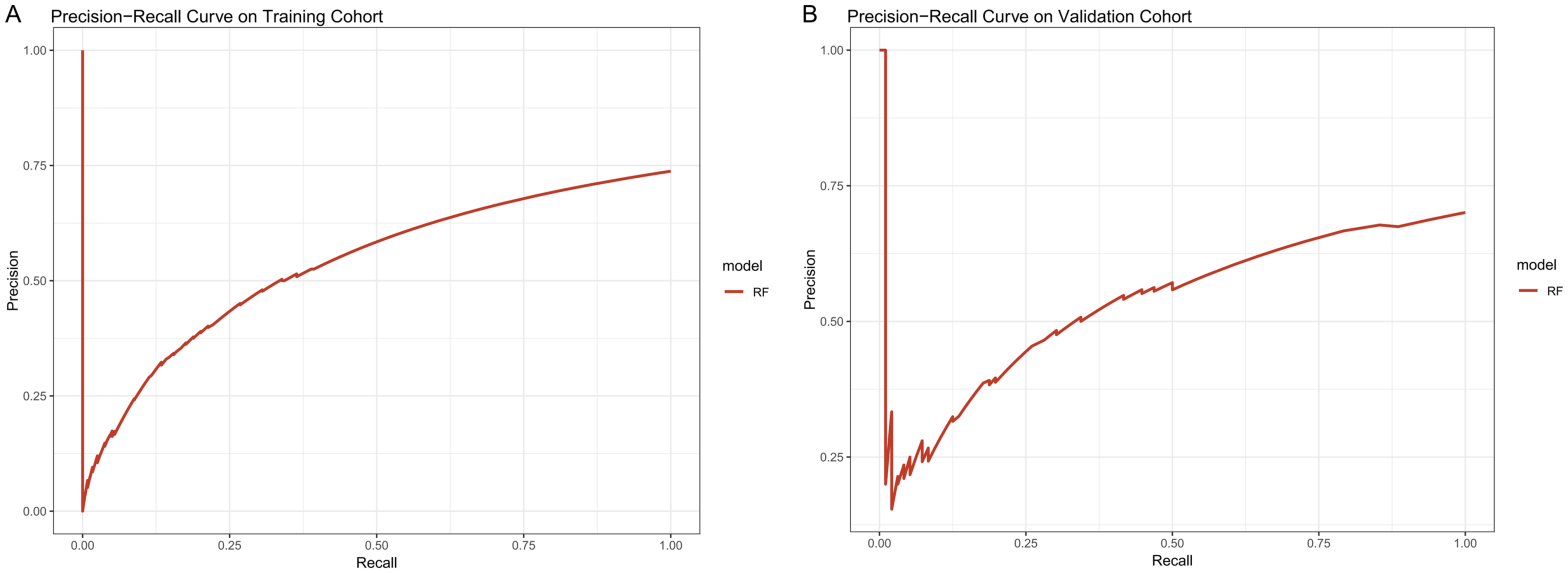
The precision recall (PR) curves of the random forest (RF) model in the training cohort **(A)** and validation cohort **(B)**.

PR curve analysis revealed that the RF model performed well in both the training and external validation cohorts (Figure 3). In the training cohort, the PR curve showed a stable trend, suggesting that the RF model consistently achieved high precision and recall across various thresholds, indicating its strong robustness. Although slight fluctuations were observed in the PR curve at very low recall levels (0.00–0.10) in the external validation cohort, the model maintained a strong classification performance within clinically relevant decision thresholds, underscoring its practical value in clinical settings.

### 3.4 Comparison of the performance of the optimal model and the Mayo score, MES, DUBLIN score, and UCEIS

**Figure 4:**
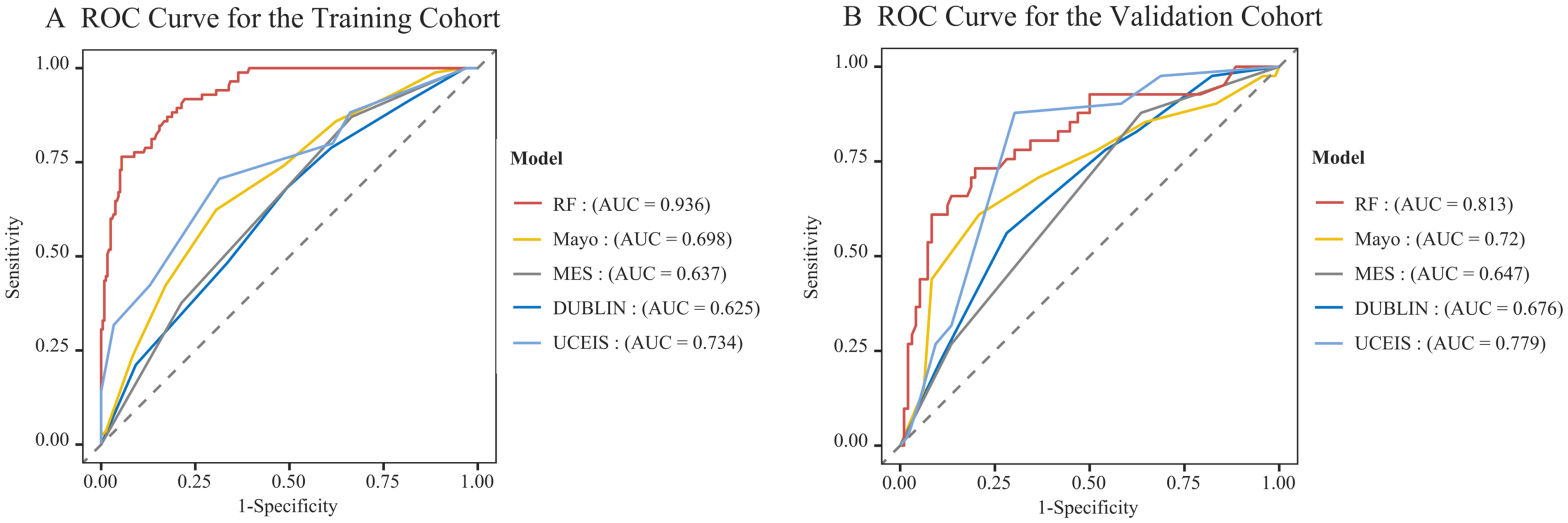
AUC comparison of the RF model and clinical scores (Mayo, MES, DUBLIN, UCEIS) for predicting unplanned readmission of UC in the training cohort **(A)** and validation cohort **(B)**. MES, Mayo Endoscopic Subscore; DUBLIN, Degree of Ulcerative Colitis Burden of Luminal Inflammation; UCEIS, Ulcerative Colitis Endoscopic Index of Severity.

Using ROC curve analysis, we compared the ability of the optimal RF model, the Mayo score, the MES, the DUBLIN score, and the UCEIS to predict unplanned readmission within 1 year (Figure 4). The results showed that the RF model achieved the best predictive performance, with AUCs of 0.936 and 0.813 in the training and validation cohorts, respectively. The UCEIS had the second-best performance, with AUCs of 0.734 and 0.779 in the training and validation cohorts, respectively. The Mayo score showed modest predictive ability in the validation cohort (AUC = 0.720), but its performance in the training cohort was relatively lower (AUC = 0.698). The predictive performance of the MES and DUBLIN scores was suboptimal, with AUCs of 0.637 and 0.625 in the training cohort and 0.647 and 0.676 in the validation cohort, respectively.

### 3.5 Feature importance and model explainability

**Figure 5:**
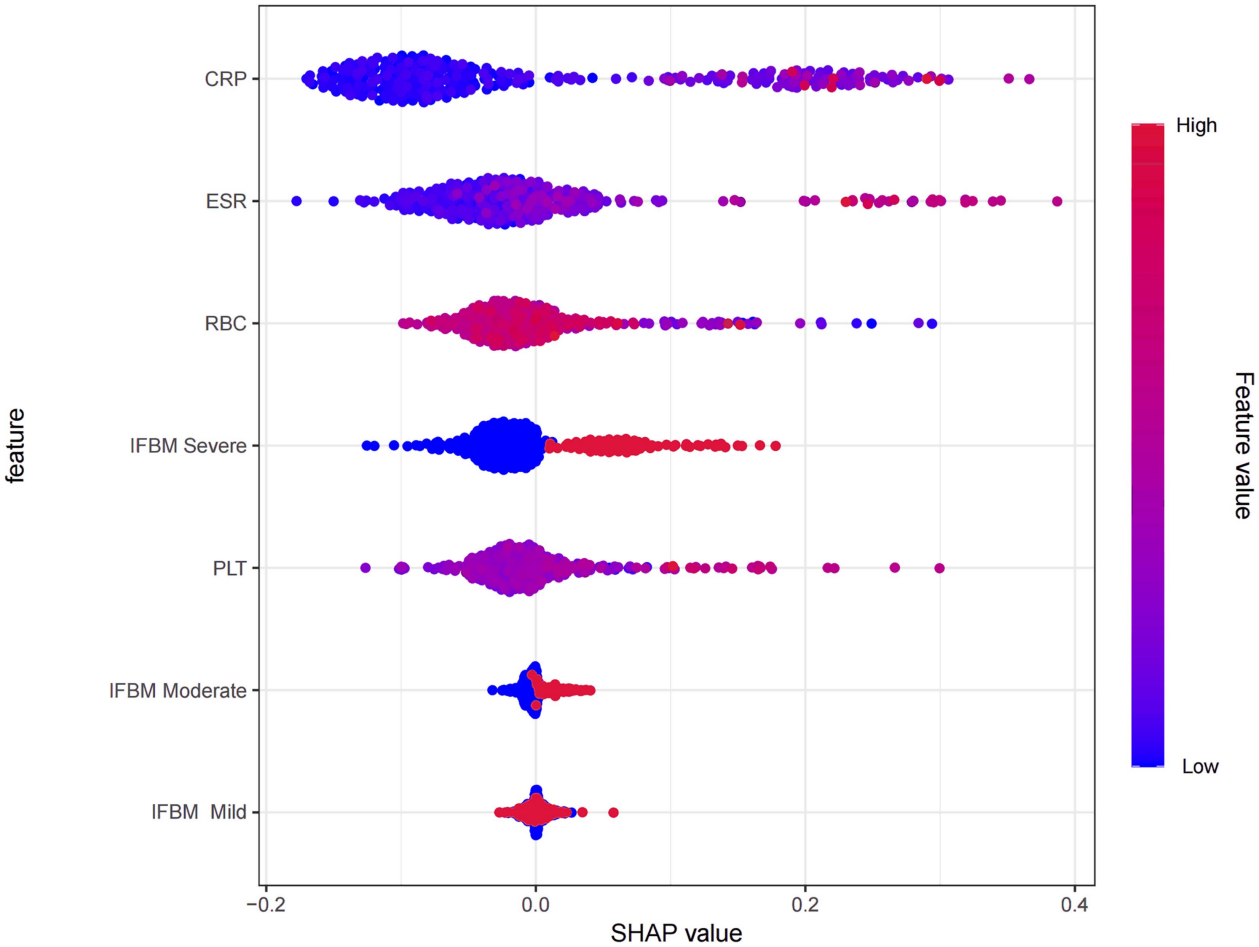
SHAP analysis of RF model used for predicting UC unplanned readmission. CRP, C-reactive protein; ESR, erythrocyte sedimentation rate; RBC, red blood cell; IFBM, increased frequency of bowel movements; PLT, platelet count.

To gain deeper insights into the decision logic of the model, SHAP plots were employed to visualize the RF model outputs (Figure 5). The top contributing features, in descending order of importance, were CRP, ESR, RBC, IFBM, and PLT. SHAP analysis revealed that CRP, ESR, IFBM Severe, PLT, and IFBM Moderate had predominantly positive SHAP values-indicating that increases in these features (as shown in red) contributed positively to predicted readmission risk and were thus identified as risk factors. In contrast, RBC and IFBM Mild had predominantly negative SHAP values, indicating that higher levels of these features were associated with a lower readmission risk and could be considered protective factors.

### 3.6 Implementation of web calculator

**Figure 6:**
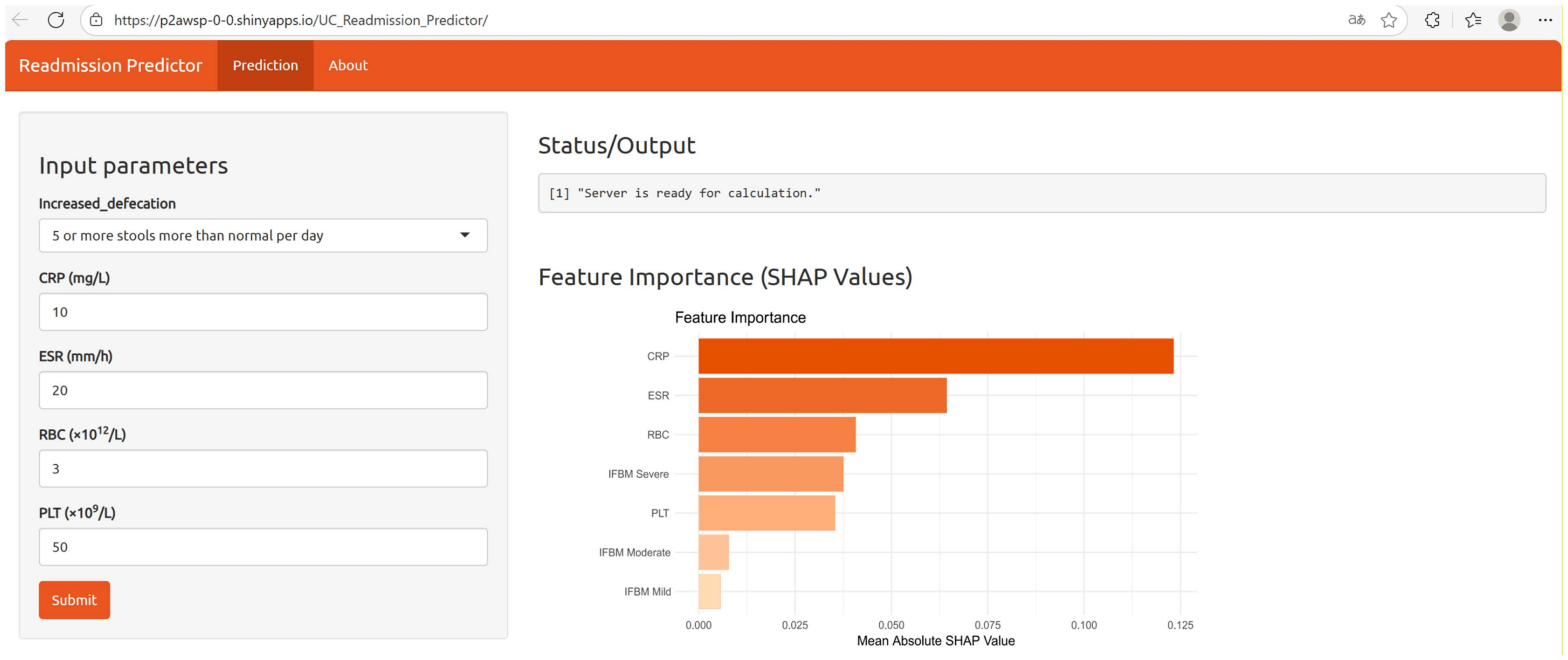
Web-based calculator for predicting unplanned readmission of UC patients.

Based on five key indicators—CRP, ESR, RBC, IFBM, and PLT—a web-based calculator was developed to provide an individualized prediction of unplanned readmission risk in patients with UC, with the goal of supporting precision management and clinical intervention^1^ (Figure 6).

## 4 Discussion

Patients with ulcerative colitis face a high risk of unplanned readmission during disease flare-ups, which presents an urgent public health concern. Unplanned readmission typically indicates worsening health status post-discharge, exacerbated clinical symptoms, increased difficulty in medication management, and heightened risks, including opportunistic infections (such as Clostridioides difficile and cytomegalovirus infections), rapid disease progression, and severe complications such as toxic megacolon, massive hemorrhage, or perforation (17). Inadequate early detection and delayed intervention may cause sudden clinical decline, elevating both mortality and long-term disability risks in some patients (18, 19). Thus, the timely identification of high-risk patients for unplanned readmissions is critical for improving clinical outcomes. However, the complex and individualized nature of UC, driven by factors such as inflammatory burden, treatment response, comorbid conditions, and psychological state, makes it difficult for even seasoned clinicians to reliably predict disease progression over a 1-year horizon. Hence, constructing a predictive model based on multidimensional clinical variables and implementing an accessible online tool is vital for reducing the risk of unplanned readmissions and optimizing the long-term prognosis of patients with UC.

This study established an ML-driven predictive platform that incorporates a wide range of clinical variables—demographics, medical history, medication profiles, symptoms, lab tests, and endoscopic findings—to estimate the 1-year unplanned readmission risk in patients with UC, enabling a holistic assessment of their clinical condition. The RF model demonstrated superior predictive performance compared with the other seven algorithms, achieving AUCs of 0.936, 0.815, and 0.813 in the training, internal validation, and 0.813 in external validation cohort, respectively. DCA results confirmed that the RF model offered a higher net clinical benefit within most clinically relevant threshold ranges, underscoring its practical utility and scalability in real-world settings. Some fluctuations were observed in the PR curve of the RF model at very low recall levels (recall <0.10) in the external validation cohort, likely due to the limited number of positive cases and uneven class distribution, which may have impacted the stability in identifying rare high-risk patients. Overall, the RF model consistently exhibited a strong classification performance across thresholds, reflecting its robustness and ability to generalize to unseen data. In summary, the online prediction platform built using the RF model demonstrated strong feasibility. Its risk prediction capabilities help patients gain clearer insights into their health status and enhance their self-management awareness through risk alerts. For high-risk patients, clinicians can intensify follow-up, promptly adjust treatment plans, and strengthen nursing monitoring. This personalized intervention not only effectively reduces unplanned readmission rates but also improves treatment outcomes and minimizes the waste of healthcare resources.

This study identified five critical predictors incorporated into the RF model: CRP, ESR, RBC, IFBM, and PLT, which represent the core clinical domains of UC, such as systemic inflammation, symptomatic severity, bleeding tendency, and nutritional condition. CRP and ESR are widely recognized markers of inflammation; their elevation reflects enhanced systemic inflammatory responses and correlates strongly with disease activity and mucosal injury (20, 21). When the intestinal mucosa is damaged, the immune system is activated, particularly local intestinal T cells and macrophages, which release inflammatory mediators (such as TNF-*α* and IL-6). This exacerbates the local inflammatory response and induces systemic acute-phase inflammatory reactions via the bloodstream, leading to elevated CRP and ESR levels (22, 23). RBCs serve as indirect indicators of persistent disease activity. In patients with UC, intestinal inflammation, malabsorption, and the release of inflammatory cytokines may lead to RBC destruction, resulting in malnutrition and anemia (24–27). As the most clinically intuitive symptom variable, IFBM reflects intestinal barrier impairment and inflammation-induced motility abnormality. An increased stool frequency is significantly associated with the risk of readmission (28, 29). PLTs are among the first cells recruited to the vascular endothelium at the sites of inflammation and infection (30). PLTs and platelet activation can trigger a cascade of inflammatory responses by increasing vascular permeability and promoting leukocyte migration, thereby exacerbating intestinal mucosal injury (31). PLTs not only serve as markers of inflammation but may also indicate the activation of the coagulation cascade, a known contributor to higher complication rates in patients with IBD (32). In summary, these five variables characterize the clinical status of patients with UC from different dimensions, providing highly representative information inputs for the model. This ensures its discriminative power and clinical interpretability for risk prediction.

In recent years, multiple studies have attempted to develop clinical indicator-based models to predict the risk of hospital readmission in patients with UC. Xiang et al. (33) developed a nomogram incorporating the AHRQ Elixhauser index, regular follow-up status, corticosteroid history, CRP level, and the UC endoscopic index of severity (UCEIS) to estimate readmission risk in UC patients. The model demonstrated an AUC of 0.764 at the 52-week follow-up in the external validation. Sobotka et al. (34) developed a logistic regression model incorporating surgical factors—including race, medication history, preoperative severity, surgery type, and postoperative complications—to predict 30-day readmission risk. In the validation cohort, the model achieved an AUC of 0.71 (95% CI: 0.66–0.75). However, these models were constrained by small sample sizes, suboptimal accuracy, and limited feature diversity, thus falling short of the requirements for personalized risk assessment. Moreover, the LR model by Sobotka et al. was not externally validated, which restricts its applicability in broader clinical settings. The RF model outperformed the existing clinical scores in both the training and validation cohorts. Mayo mainly focuses on disease activity (35). MES is the endoscopic component of the Mayo score and is simpler (36). UCEIS assesses endoscopic severity in three domains: vascular pattern, bleeding, and erosions/ulcers (37). DUBLIN scores is mainly used to quantify the inflammatory burden of ulcerative colitis (38). However, the lack of integration of comprehensive clinical data in these scores limits their ability to predict multifactorial outcomes, such as readmission. In contrast, the RF model integrates diverse clinical features to provide a comprehensive view of patient status, thereby improving its capacity to accurately discriminate high-risk readmission.

Our study has several strengths. First, we conducted a comprehensive screening of multidimensional clinical variables to identify key predictors, minimizing the potential omission of relevant features and enhancing the model’s predictive accuracy. Second, the model was externally validated using a prospectively collected independent cohort, confirming its satisfactory applicability in clinical practice. Third, the optimal model was selected by comparing the performance of multiple ML algorithms, and an online risk prediction platform was developed based on the top-performing RF model. This web-based risk calculator is freely available to the public, providing clinical decision support for physicians in assessing readmission risk, while also aiding patients in scheduling follow-ups and improving self-management. Looking forward, we will explore integration of this tool into electronic health record systems to enable automated data transfer and real-time risk updates, thereby enhancing its clinical utility in real-world settings.

### 4.1 Limitations and prospects

However, this study had certain limitations. First, this was a single-center study. Although the applicability of the model was preliminarily validated in a prospective cohort, multicenter prospective studies are still needed to further assess its external generalizability. Second, the model was developed using retrospective data. Although multiple clinical dimensions were considered, the early initiation of the study led to high missing rates in fecal calprotectin, other histologic indices, and psychosocial variables, which prevented some potentially informative predictors from being included in the model. To address this limitation, we have designed a new prospective cohort study using predesigned case report forms and standardized data collection procedures, in which the above variables will be systematically incorporated into predictor selection and model development, with the aim of further improving model performance. Third, the study population consisted only of hospitalized patients with UC, and it remains unclear whether the model is applicable to UC management in the outpatient setting. In the future, we plan to integrate the RF model more broadly into the clinical decision-making process for patients with UC and to continuously accumulate case data across different clinical contexts in order to further evaluate and optimize its predictive performance.

## 5 Conclusion

This study established a RF model based on five clinically accessible predictors—CRP, ESR, RBC, IFBM, and PLT—with reliable predictive performance for 1-year outcomes in patients with UC. An online platform was developed based on the RF model to assist clinicians in performing individualized risk assessments, thereby optimizing treatment strategies and reducing the risk of unplanned readmission.

## Data Availability

The raw data supporting the conclusions of this article will be made available by the authors, without undue reservation. Requests to access these datasets should be directed to the first author, Tianqi Wang, wwwsunlight77@163.com.

## Footnotes

1
https://p2awsp-0-0.shinyapps.io/UC_Readmission_Predictor/
