# Supplementary material for: Development and evaluation of a machine learning model to predict unplanned readmission risk in patients with ulcerative colitis
Source: Front Med (Lausanne). 2026 Jan 27;13:1712846. doi: 10.3389/fmed.2026.1712846 (PMC12886491; doi:10.3389/fmed.2026.1712846)
Supplement: Supplementary file 1 [file Supplementary_file_1.docx]

Supplementary Material

Development and Evaluation of a Machine Learning Model to Predict Unplanned Readmission Risk in Patients with Ulcerative Colitis

**Supplementary figure 1.** Feature selection using Recursive Feature Elimination.


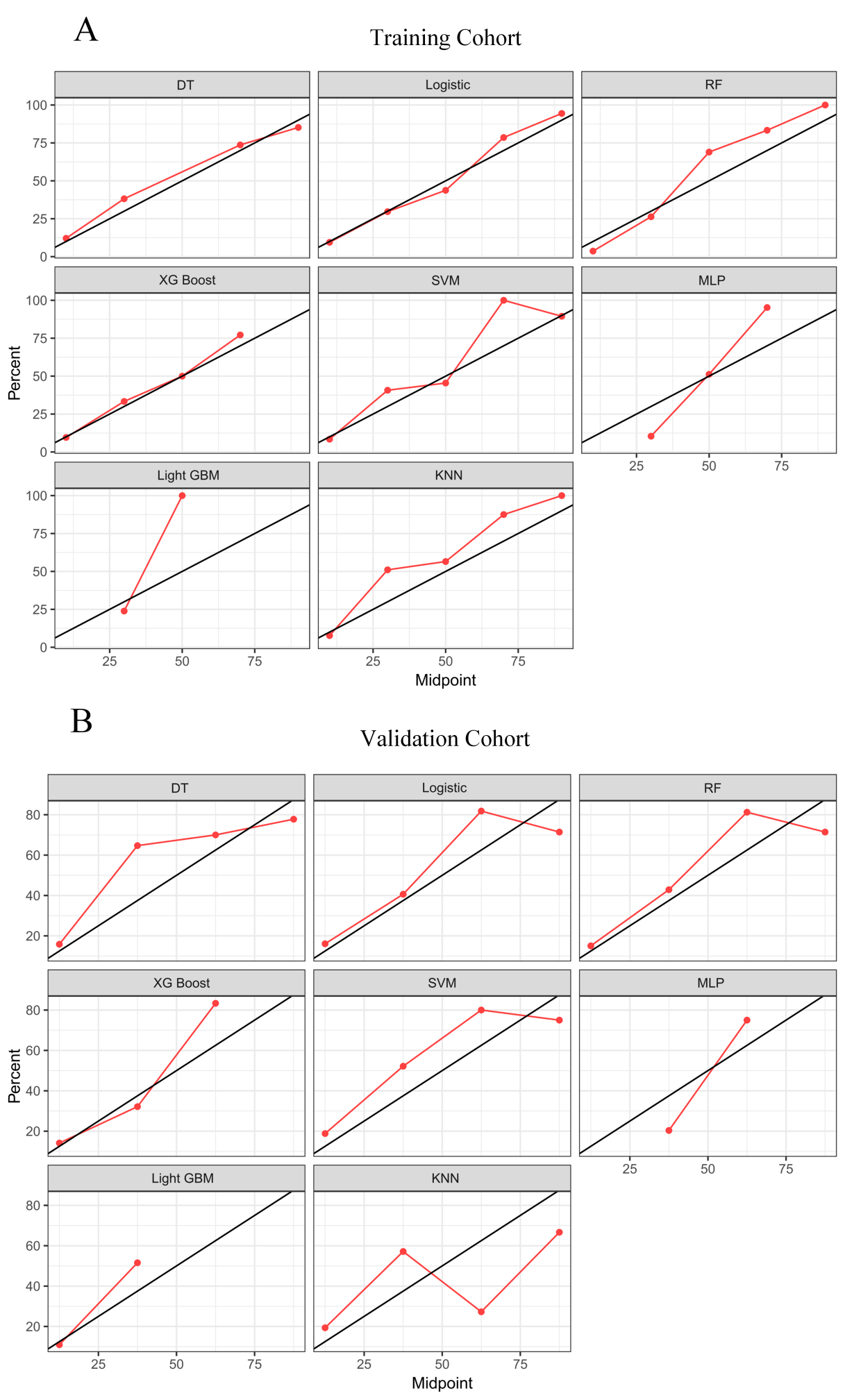


**Supplementary figure 2.** Calibration curves of eight machine learning models in (A) the Training cohort and (B) the Validation cohort.

**Abbreviations:** DT: decision tree; RF: random forest; XG Boost: extreme gradient boosting; SVM: support vector machine; MLP: multilayer perceptron; Light GBM: light gradient boosting machine; KNN: k-nearest neighbors.

**Supplementary table 1** Fecal Calprotectin Data

| Fecal calprotectin (µg/g) | With unplanned readmission | | Without unplanned readmission | | *P-*value | Missing rate (%) |
| --- | --- | --- | --- | --- | --- | --- |
|  | N | Median (Q1, Q3) | N | Median (Q1, Q3) |  |  |
| All data | 68 | 453.87 (93.24, 600.00) | 203 | 294.18 (52.00, 600.00) | 0.063 | 41.2 |
| Training cohort | 35 | 600.00 (89.31, 600.00) | 117 | 232.29 (51.15, 600.00) | 0.118 | 53.1 |
| Validation cohort | 33 | 378.60 (112.04, 600.00) | 86 | 304.70 (70.62, 600.00) | 0.312 | 13.1 |

Fecal calprotectin values > 600 μg/g were reported as “ > 600 μg/g ” due to the upper detection limit of the assay at our center.
